# The splenic response to stroke: from rodents to stroke subjects

**DOI:** 10.1186/s12974-018-1239-9

**Published:** 2018-07-03

**Authors:** Hilary A. Seifert, Halina Offner

**Affiliations:** 1 Neuroimmunology Research R&D-31 Veterans Affairs Portland Health Care System, 3710 SW US Veterans Hospital Rd, Portland, OR 97239 USA; 2 Department of Neurology, Oregon Health and Science University, Portland, OR USA; 3 Department of Anesthesiology and Perioperative Medicine, Oregon Health and Science University, Portland, OR USA

**Keywords:** Stroke, Splenectomy, Immune response, Clinical studies, Animal models

## Abstract

**Background:**

Stroke is the fifth leading cause of death and the leading cause of long-term disability in the USA, costing $40.2 billion in direct and indirect costs. Globally, stroke is the second leading cause of death and has a higher prevalence in lower- and middle-income countries compared to high-income countries.

The role of the spleen in stroke has been studied in rodent models of stroke and is seen as a major contributor to increased secondary neural injury after stroke. Splenectomy 2 weeks prior to ischemic and hemorrhagic stroke in mice and rats shows decreased infarct volumes. Additionally, the spleen decreases in size following stroke in rodents. Pro-inflammatory mediators are also increased in the spleen and subsequently the brain after stroke. These data in preclinical models of stroke have led stroke neurologists to look at the splenic response in stroke subjects. The outcomes of these studies suggest the spleen is responding in a similar manner in stroke subjects as it is in animal models of stroke.

**Conclusion:**

Animal models demonstrating the detrimental role of the spleen in stroke are providing strong evidence of how the spleen is responding during stroke in human subjects. This indicates treatments targeting the splenic immune response in animals could provide useful targets and treatments for stroke subjects.

## Background

Stroke is the leading cause of disability and the fifth leading cause of death in the USA. Each year, 42.4 million strokes occur globally. The most common type is ischemic stroke resulting from a clot occluding a cerebral vessel [1]. While advancements have been made in treating the clot and restoring blood flow to the brain, there are still many factors that influence infarct size and the resulting disability. The immune system has been shown to play a detrimental role after stroke [2–4]. The spleen is a storage site for immune cells [5] and has been shown in animal models to play a role in increasing neural injury after experimental stroke [6–10]. This review will examine the spleen’s role in animal models of stroke and how these studies have translated into examining the spleens in stroke subjects.

### Stroke statistics

Stroke is the fifth leading cause of death in the USA and second leading cause of death around the world. In the USA, 795,000 strokes occur annually resulting in the largest cause of long-term disability and costing $40.1 billion in direct and indirect costs. Globally 42.4 million strokes occur each year, with high-income countries seeing a decrease in the incidence of ischemic and hemorrhagic stroke compared to low and middle income countries. Of the two main stroke subtypes, ischemic and hemorrhagic, ischemic strokes account for 87% of strokes while hemorrhagic strokes make up 13%. There are racial differences in stroke risks and outcomes. American Indian/Alaska Natives and non-Hispanic blacks have higher stroke prevalence compared to non-Hispanic whites, Hispanics, and Asians/Pacific Islanders (~ 4.5 vs. ~ 2%, respectively). Additionally there are differences between men and women with regards to stroke risk, incidence, and outcomes. Overall women have a higher lifetime risk of stroke than men, and 55,000 more women than men experience a stroke each year. Men are at a slightly higher risk for stroke in younger and middle-aged groups but women have a much greater risk for stroke later in life and are more likely to die from a stroke. Women accounted for 58% of stroke deaths in the USA in 2015 [1].

### The spleen

The spleen is a secondary immune organ that is part of the immune system. The spleen acts as a filter for the blood to remove any dying red blood cells, antibody coated cells, and bacterial pathogens. Additionally, the spleen serves as a communication hub for various immune cells to interact with each other, including T cells, B cells, macrophages/monocytes, and dendritic cells to initiate adaptive immune responses. The spleen is divided between the red and white pulp. The red pulp contains macrophages that filter the blood as it passes through the spleen and antibody producing B cells that allow for rapid delivery of antibodies into circulation. The white pulp has concentrated areas of lymphocytes. The close proximity of these cells allows for easy interaction of T cells and B cells to mount adaptive responses and allows macrophages and dendritic cells easy access to lymphocytes. T cells are concentrated around arterioles and make up periarteriolar lymphoid sheaths. This allows T cells to be in close proximity to the blood supply that allows dendritic cells easy access to naïve T cells, which once activated leave the spleen. B cells are organized to form B cell follicles. B cell follicles are where B cell responses are honed with isotype switching and increasing antigen specificity. These processes require T cells. The highly specific structure of the spleen allows for all these cells to be in close proximity to each other [5].

### Peripheral immune response to stroke

Numerous studies have demonstrated the role the immune system plays in increasing neural injury follow stroke. Mice lacking lymphocytes, both Rag^−/−^ [2] and SCID mice [3], have decreased infract volumes compared to wild type mice following middle cerebral artery occlusion (MCAO). An additional study showed peripheral immune cells in the stroked brain hours to days after the initial insult [4]. The spleen, which is a reservoir of immune cells, is of interest in secondary neural injury following stroke. Splenocytes have been found in the brain in only the injured hemisphere at 48 and 96 h following MCAO [11], a rodent model of stroke.

### Splenic response to ischemia/reperfusion injuries

The spleen’s involvement is well documented to enhance inflammation in ischemia/reperfusion (IR) injuries to various organs. The liver was the first organ where splenectomy was shown to decrease injury from the IR and blunt the inflammatory response triggered by IR [12]. Splenectomy also protected the intestines [13] and the kidneys [14] from IR injury. The injury in all three organs is believed to be mediated by monocytes in the spleen that activate Kupffer cells in the liver. Removing the spleen and splenic monocytes prevents activation of Kupffer cells, resulting in less inflammation and a reduction in tissue damage, as does blocking Kupffer cell activation with gadolinium chloride. Splenic monocytes were also found to play a detrimental role following IR injury to the heart, since splenectomy was protective against IR injury in that organ [15].

### Splenic response to stroke in rodents

Rodent spleens show a decrease in size after MCAO that appears to be catecholamine (CA)-mediated, but the reason for the change in spleen size after stroke varies between rats and mice. The splenic capsule of rat spleens expresses α_1_ adrenergic receptors that cause splenic contraction when activated [16]. Following permanent MCAO (pMCAO) in rats, circulating CAs cause a transient decrease in spleen size. While the spleen is highly innervated by sympathetic neural networks [17], denervation of the spleen prior to pMCAO did not prevent the spleen from contracting. However, blocking the α_1_ receptors with prazosin or carvedilol did block splenic contraction, whereas propranolol did not block contraction. Carvedilol also decreased infarct size [18]. Additionally, rat spleens demonstrated a transient decrease in size with the smallest size detected 48 h after pMCAO but a return to pre-stroke size by 72 h. During splenic contraction, splenocytes were released into the systemic circulation and traveled to the injured hemisphere of the brain. Innate splenocytes were found in the brain as early as 48 h, whereas adaptive splenocytes appeared at 96 h following pMCAO [11]. Generally, there was a negative correlation between spleen size and infarct volume [19]. Mice also exhibit a significant loss of cells and decreased spleen size following transient MCAO (tMCAO) that persists over time. This loss of splenocytes appears to be due to apoptosis of the splenocytes and loss of splenic structures, including B cell follicles. At 96 h after tMCAO, mice show a 90% reduction in splenocyte numbers compared to sham-operated mice. The greatest effect is on B cells, while T cell numbers appear to increase, specifically regulatory T cells [20]. An additional study showed that mouse spleens continued to decrease in size through day 7 post-tMCAO [21]. Mouse spleens are extensively innervated like rat spleens and also appear to highly express α_1_-adenergic receptors on the splenic capsule [22]. Unlike rats, mouse spleens do not contract, but the catecholaminergic innervation of immune cells appears to negatively impact some of these cells resulting in cell death.

In addition to the cellular responses in the spleen to stroke, there are also changes in cytokine and chemokine responses. In mouse spleens, there is an increase by 22 h after tMCAO in TNFα, interferon gamma (IFNγ), IL-6, MCP-1, and IL-2, all of which are pro-inflammatory mediators [23]. One pro-inflammatory cytokine of interest is IFNγ, since IFNγ^−/−^ mice have smaller infarcts than wild-type mice [2]. In rats, splenic increases in IFNγ production as early as 24 h after pMCAO results in a delayed increase in IFNγ in the injured brain at 72 h. This early transient increase in IFNγ in the spleen is likely connected to the later increase in IFNγ in the brain, since splenectomy decreases the IFNγ expression in the brain [7]. Similar results were found in mice after tMCAO such that IFNγ was increased at 72 h in the brain and splenectomy significantly decreased IFNγ levels within the brain [8]. IFNγ also initiated the expression of the pro-inflammatory chemokine interferon inducible protein 10 (IP-10) in both the spleen and the brain after pMCAO. Splenectomy prior to pMCAO or treatment with an IFNγ neutralizing antibody after pMCAO reduced IP-10 levels in the brain and subsequent T cell recruitment to the brain [24].

### Splenectomy in rodent models of stroke

Splenectomy carried out 2 weeks prior to initiating MCAO in mice or intracerebral hemorrhage (ICH) in rats afforded protection in the brain. Initial studies found that splenectomy 2 weeks prior to pMCAO in rats decreased infarct volume by 80% and reduced immune cell infiltration into the brain [6]. Subsequently, a study found that splenectomy significantly decreased edema and water content in a rat model of ICH [10]. In mice, splenectomy prior to tMCAO was first carried out only in males and showed a significant reduction in infarct volume [8]. Additional studies in mice again showed that male mice were protected from stroke after splenectomy but that the protective effect was lost in female mice [9]. It was found that female mice had more regulatory lymphocytes in their spleens than male mice and the loss of these regulatory cells in female mice resulted in the loss of protection from stroke [9, 25]. Additionally, male mice had more activated T cells in their spleens after tMCAO compared to female mice [26]. A study in older mice demonstrated that splenectomy decreased infarct volume in older male mice, improved behavioral outcomes, and decreased immune cell infiltration into the brain [27]. A more recent study in rats showed splenectomy immediately following tMCAO significantly decreased infarct volume and improved behavioral deficits [28]. Other studies have concluded that splenectomy does not protect mice or rats from tMCAO although it was noted that the MCAO surgery was performed immediately after the splenectomy surgery [29, 30]. Splenectomy is a major surgery, and the body compensates for the loss of the spleen by transiently increasing circulating immune cells over the course of a week (data not published). Additionally, the body is also responding to the surgical insult of removing the spleen. Therefore, splenectomy should be performed 2 weeks prior to the MCAO surgery to allow the animal’s body to recovery from the splenectomy surgery. Another study investigating the role the spleen plays in enhancing neural injury used splenic irradiation in place of splenectomy as a noninvasive way to blunt the splenic responses. Rats received targeted splenic irradiation 3 or 4 h after tMCAO, and this significantly decreased infarct size but did not damage the spleen or surrounding organs or lead to immune suppression or increased infections [31].

### Neuroprotective therapies also involve the spleen

Various therapies that protect the brain after stroke interact with the spleen. Some therapies interact with splenocytes or travel to the spleen. Others prevent the decrease in spleen size seen after tMCAO. Stem cells from various sources protect the brain after stroke and also travel to the spleen. Human umbilical cord blood (HUCB) cells reduce infarct after pMCAO in rats and restore spleen size at 48 h post-pMCAO [19, 32]. HUCB cells protected gray and white matter [33] in rats when administered 24 h after MCAO and reduced behavioral deficits. Additionally, HUCB cells saved the spleen weight [19], were found in the injured brain and the spleen [34], and allowed splenocytes to respond normally when stimulated with concanavalin A when the cells were administered intravenously [19]. Studies with human bone marrow stem cells (hBMSC) found these cells decreased infarct volume in rats and migrated to the spleen when administered intravenously [35]. The previous two examples were immune cell derived stem cells that migrated to the spleen after stroke to help reduce the infarct. A study in a model of ICH used neural stem cells and found these cells were more effective at reducing edema and brain water content after ICH when injected intravenously compared to direct injection into the brain. In addition, it was reported that neural stem cells in the spleen were in direct contact with the splenic monocytes. The spleen was also found to be necessary for neural stem cells to provide protection since protection was lost when the rats underwent splenectomy 2 weeks prior to ICH and neural stem cell administration [10].

Other therapies save spleen size and reduce infarct. One such therapy is treatment with recombinant T cell receptor ligands (RTL), which are partial major histocompatibility complex II molecules covalently bound to myelin peptides. After tMCAO in mice, RTL administration reduced infarct size by 50%, reduced the recruitment of immune cells to the brain, and prevented the reduction in spleen size seen in mice. This effect was only seen in mice given the RTL with a myelin peptide [36–38]. Another therapy, carvedilol—a pan adrenergic blocker, also reduced infarct size in rats with pMCAO and prevented splenic contraction [18].

### The splenic response in stroke subjects

The initial pilot study in stroke subjects used ultrasound to evaluate at the spleen size in 30 stroke subjects with ischemic stroke that were at the hospital within 6 h after onset of symptoms. This study found that the spleen size of subjects initially decreased and if the subject continued to decline on the NIH Stroke Severity (NIHSS) score then their spleens continued to decrease in size. However, in some subjects, the NIHSS score decreased as the spleen size began to increase until it surpassed the initial ultrasound reading [39]. Another study used a retrospective approach looking at CT scans of 82 stroke subject spleens. This study included both ischemic and hemorrhagic stroke subjects. The study concluded that the spleen undergoes a biphasic response to stroke characterized by a transient decrease in spleen size reaching its smallest size at 48 h post stroke. There were gender differences in spleen size after stroke, but no difference between stroke subtypes [40]. A larger study derived from the initial pilot study was expanded to include both ischemic and hemorrhagic stroke subjects and a cohort of healthy control subjects to establish a baseline spleen size. The 158 healthy controls underwent an ultrasound of their spleen for 5 consecutive days. Based on these data, significant differences were found between gender, height, weight, and body surface area (BSA). To evaluate if there was any splenic contraction in stroke subjects, their spleen volumes were compared to healthy controls based on gender and BSA. A spleen was considered to be contracted if it was 20 cm^3^ smaller than their gender and BSA matched healthy controls. This study found that 40.5% of stroke subjects had a contracted spleen. Individuals were at a significantly higher risk for splenic contraction if they were African American, over the age of 75, had a more severe stroke, or had a history of stroke. There were no differences between genders, types of stroke, or admission NIHSS score. Plasma cytokines were also analyzed in a subset of subjects and individuals with splenic contraction had significantly elevated levels of IFNγ, IL-6, IL-12, and IL-10 [41]. In an expansion of the previous study, a retrospective looked at only ischemic stroke subjects to determine if there was an association between splenic contraction and systemic inflammatory response syndrome (SIRS). SIRS is an inflammatory process that is detrimental to stroke recovery and independent of an infection. SIRS was considered present if two of the following were present: elevated heart rate, changes in body temperature, elevated respiratory rate, or changes in white blood cell counts. Non-infectious SIRS is associated with longer hospital stays and poorer functional outcomes. Splenic contraction was not associated with the development of early SIRS. African Americans and individuals aged over 75 were at a significantly higher risk of splenic contraction following a stroke. African Americans had a 92.3% chance of developing SIRS within the first 48 h of stroke if they had splenic contraction compared to a 62.5% chance of developing SIRS with splenic contraction in other races. In individuals over the age of 75, there was a negative correlation with splenic contraction and the development of SIRS at 72 h post stroke, while there was no significant connection between splenic contraction and SIRS in the younger age groups. In stroke subjects over the age of 75, the development of SIRS was higher in individuals without splenic contraction compared to those subjects with splenic contraction (71.4 vs. 46.1, respectively) [42].

## Conclusion

**Fig. 1:**
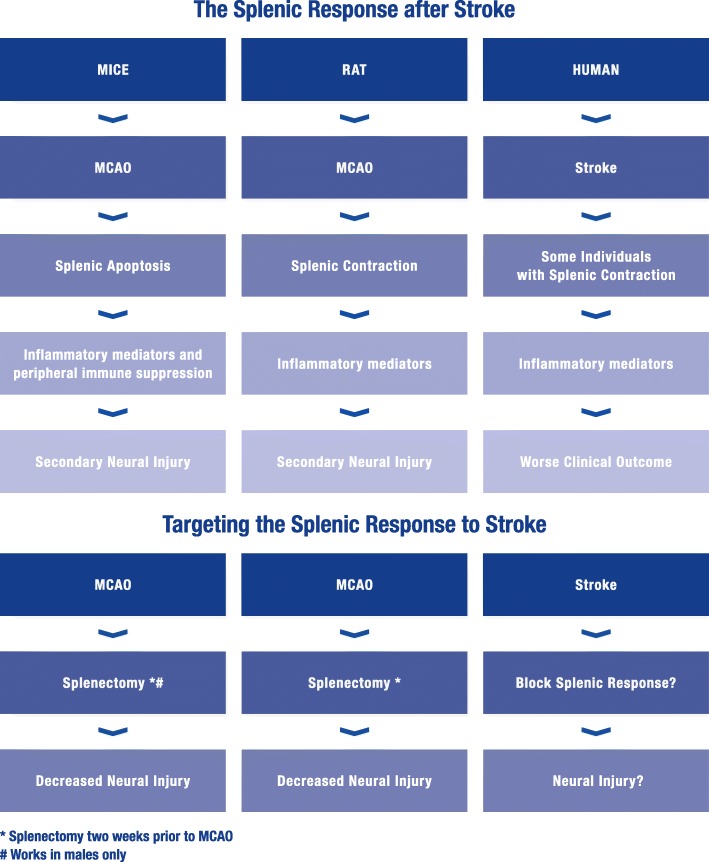
The splenic immune response to stroke. The spleen responds to stroke in mice, rats, and humans after cerebral ischemia. In experimental models with mice and rats using MCAO as a model for ischemic stroke, the spleen decreases in size. In mice, this is due to splenic apoptosis and loss of cells. In rats, the spleen contracts and releases immune cells into circulation. In stroke subjects, the spleen contracts in some individuals. The spleen’s involvement in post-stroke events contributes to secondary neural injury and worse outcomes. When the spleen is targeted in animal models of stroke, using splenectomy, this decreases neural injury in mice and rats. Can targeting the spleen in humans improve clinical outcomes by decreasing neural injury? * indicates splenectomy 2 weeks prior to MCAO. # splenectomy only works in male mice

The importance of the spleen in contributing to increased neural damage following a stroke is becoming increasing evident in stroke subjects. Changes that were seen in preclinical models of stroke in rodents are similar to the data being collected in stroke subjects. While there are limitations to all animal models of human diseases, the role of the spleen in stroke from preclinical models so far has provided some insight into the disease process of human stroke. Not only has the spleen been shown to decrease in size following stroke there is also an increase in IFNγ in subjects with splenic contraction (Table 1). This could lead to biomarkers for stroke outcomes or targets to treat stroke in certain subsets of subjects (Fig. 1).

**Table 1:** Studies looking at the splenic response to stroke

		Species	Type	Duration	Citation
Observations	Splenocyte apoptosis	Mus	tMCAO	96 h	[20]
	Increased IFNg levels	Mus	tMCAO	96 h	[8]
		Rat	pMCAO	96 h	[7, 24]
	Splenic contraction	Rat	pMCAO	96 h	[7]
	Change in spleen size	Hu	Ischemic	Variable	[39]
		Hu	Ischemic hemorrhagic	Variable	[40]
	Splenic contraction (some subjects)	Hu	Ischemic hemorrhagic	Variable	[41]
	SIRS with splenic contraction (some subjects)	Hu	Ischemic	Variable	[42]
Pre-treatment	Splenectomy protection (males only)	Mus	tMCAO	96 h	[9]
	Splenectomy protection	Mus	tMCAO	96 h	[8]
		Rat	pMCAO	96 h	[6]
		Rat	ICH	72 h	[10]
	Carvedilol	Rat	pMCAO	48 h	[18]
Post-treatment	RTL	Mus	tMCAO	96 h	[36–38]
	Splenic irradiation	Rat	tMCAO	48 h, 7 days	[31]
	HUCBC’s	Rat	pMCAO	48 h, 96 h	[19, 32–34]
	NSC	Rat	ICH	72 h	[10]

This table summarizes experiments that involve the splenic response in stroke. Some observational studies, some pre-treatment studies, some post-treatment studies. Studies with mice, rats, and human stroke subjects are included

## Abbreviations

BSA: Body surface area
CA: Catecholamine
hBMSC: Human bone marrow stem cells
HUCB: Human umbilical cord blood
ICH: Intracerebral hemorrhage
IFNγ: Interferon gamma
IP-10: Interferon inducible protein 10
IR: Ischemia/reperfusion
MCAO: Middle cerebral artery occlusion
NIHSS: NIH stroke severity
NSC: Neural stem cells
pMCAO: Permanent MCAO
RTL: Recombinant T cell receptor ligands
SIRS: Systemic inflammatory response syndrome
tMCAO: Transient MCAO
